# COMBINED USE OF NOVEL MR IMAGING AND FLUID BIOMARKERS REVEAL DETERMINANTS OF MYELIN AND AXONAL LOSS WITH AGING

**DOI:** 10.1093/geroni/igad104.1279

**Published:** 2023-12-21

**Authors:** Keenan Walker

**Affiliations:** National Institute on Aging, National Institutes of Health, Baltimore, Maryland, United States

## Abstract

Cerebral white matter damage is a feature of Alzheimer’s disease, vascular dementia, and other neurodegenerative conditions, yet little is known about the molecular underpinnings of key features of white matter pathology. In a series of studies we have used targeted assays and large-scale proteomics platforms to determine how biomarkers of Alzheimer’s disease pathology (Aß42/40 ratio; pTau181), neuronal injury (NfL), reactive astrogliosis (GFAP), and other disease processes relate to state-of-the-art diffusion-based MRI measures of brain myelin content and axonal density in cognitively unimpaired older adults. Using data from the Baltimore Longitudinal Study of Aging (BLSA) and the GESTALT study, we measured plasma biomarkers using the Quanterix Simoa assay (targeted measurement of 4 proteins) and the SomaScan proteomic platform (untargeted measurement of 7000 proteins). Additionally, participants underwent 3T MRI, from which measure of myelin content (defined by myelin water fraction [MWF]) and axonal density (defined by neurite density index [NDI]) were derived. We found an association between higher plasma GFAP and lower myelin content in temporal brain regions. Additionally, higher levels of NfL and GFAP were associated with lower total brain axonal density. Using untargeted proteomics, we identified a set of 155 plasma proteins that were associations with myelin content (P <0.05). Using these findings, we applied machine learning to construct a plasma protein-based predictor of brain myelin content, which we plan to validate in a cohort of individuals with multiple sclerosis: a condition characterized by demyelination.

